# Comparative assessment of reported symptoms of influenza, respiratory syncytial virus, and human metapneumovirus infection during hospitalization and post‐discharge assessed by Respiratory Intensity and Impact Questionnaire

**DOI:** 10.1111/irv.12903

**Published:** 2021-09-02

**Authors:** Ann R. Falsey, Edward E. Walsh, Richard H. Osborne, Yannick Vandendijck, Xiaohui Ren, James Witek, Diye Kang, Eric Chan, Jane Scott, Gabriela Ispas

**Affiliations:** ^1^ School of Medicine Rochester Regional Health University of Rochester Rochester New York USA; ^2^ Department of Health and Medical Sciences Swinburne University of Technology Melbourne Victoria Australia; ^3^ Janssen Infectious Diseases Beerse Belgium; ^4^ Janssen Research & Development, LLC Titusville New Jersey USA; ^5^ Janssen Global Services, LLC Raritan New Jersey USA

**Keywords:** global prospective study, hMPV, influenza, patient‐reported outcomes, RiiQ™, RSV

## Abstract

**Background:**

The hospitalized acute respiratory tract infection (HARTI) study used the Respiratory Intensity and Impact Questionnaire (RiiQ™) Symptom Scale, derived from FluiiQ™, to assess and compare the burden of respiratory infection symptoms for patients with influenza, respiratory syncytial virus (RSV), and human metapneumovirus (hMPV) infection, with or without core risk factors (CRF) (age ≥65; chronic heart, renal, obstructive pulmonary disease; asthma).

**Methods:**

This was a prospective cohort study in adult patients hospitalized with acute respiratory tract infection (40 centers, 12 countries) during two consecutive influenza/RSV/hMPV seasons (2017–2019). The RiiQ™ Symptom Scale and EuroQol 5‐Dimensions 5‐Levels (EQ‐5D‐5L) were assessed by interview at two timepoints during hospitalization and at 1, 2, and 3 months post‐discharge.

**Results:**

Mean lower respiratory tract (LRT) symptom scores were higher for RSV and hMPV participants compared to influenza at 48 h after enrollment/early discharge (*p* = 0.001) and 3 months post‐discharge (*p* = 0.007). This was driven by LRT symptoms, including shortness of breath (SOB) (*p* < 0.01) and wheezing (*p* < 0.01) during hospitalization, and SOB (*p* < 0.05) and cough (*p* < 0.05) post‐discharge. Participants with CRF reported more moderate‐to‐severe SOB (*p* < 0.05) and wheezing (*p* < 0.05) compared to CRF(−) participants post‐discharge. EQ‐5D‐5L scores were moderately associated with RiiQ™ LRT and systemic symptoms domains.

**Conclusions:**

Results from the HARTI study suggest that in the study population, LRT symptoms were more severe for RSV and hMPV groups and for patients with CRF. RiiQ™ Symptom Scale scores shows a moderate association with EQ‐5D‐5L indicating that the RiiQ™ may provide useful insights and offer advantages over other measures for use in interventional RSV adult clinical studies.

## 1 INTRODUCTION

Patient‐reported outcomes (PROs) play an important role in assessing symptom severity and impact of respiratory pathogens, as well as in exploring the efficacy and effectiveness of prophylactic and/or therapeutic agents. The few well‐tested PRO measures available to evaluate the symptoms of influenza include the Influenza Intensity and Impact Questionnaire (FluiiQ™) Symptom Scale and the inFLUenza Patient‐Reported Outcome instrument (FLU‐PRO).^1^, ^2^ Respiratory syncytial virus (RSV) causes acute respiratory tract infection (ARTI) that presents with similar symptoms to influenza and results in substantial morbidity and mortality, particularly among older adults aged ≥65 years; however, there is limited evidence of symptom burden associated with RSV in adults.^3^, ^4^, ^5^ PROs that are designed for influenza are often used or adapted for patients with RSV and other ARTIs.^2^, ^6^, ^7^ A comprehensive symptom assessment that robustly measures the burden of RSV infection in both inpatient and outpatient settings would be an important tool for clinical research in RSV.

The Respiratory Intensity and Impact Questionnaire (RiiQ™, Measured Solutions for Health P/L, Australia) is a PRO tool used to measure symptoms (respiratory and systemic) and impact of RSV infection. Data from a phase 2b RSV vaccine study and from two observational studies in hospitalized RSV patients demonstrated initial evidence of the reliability, validity, and responsiveness of the RiiQ™ Symptom Scale and its domains. In particular, the LRT domain demonstrated strong psychometric properties with moderate‐to‐strong correlations observed between the LRT domain, clinician‐reported signs/symptoms scores and patient global impression of severity. This supports construct validity and consistency between clinician and patient ratings.^8^

The hospitalized acute respiratory tract infection (HARTI) study evaluated the distribution of influenza, RSV, and human metapneumovirus (hMPV) in patients hospitalized for ARTI and included a prospective substudy, which aimed to describe disease burden of influenza, RSV, and hMPV infection assessed by clinicians and patients during hospitalization and up to 3 months post‐hospital discharge. The RiiQ™ Symptom Scale was used to evaluate patient‐reported symptom severity of RSV, hMPV, and influenza infection. This manuscript provides a descriptive assessment of the RiiQ™ Symptom Scale and EuroQol 5‐Dimensions 5‐Levels (EQ‐5D‐5L) Health Status Questionnaire collected in patient interviews in the HARTI substudy. HARTI includes unique information on the comparative assessment of symptoms associated with three of the most important respiratory viral pathogens (influenza, RSV, and hMPV) and quantitative information on disease severity in presence of risk factors.

## 2 METHODS

### 2.1 Study design

**FIGURE 1:**
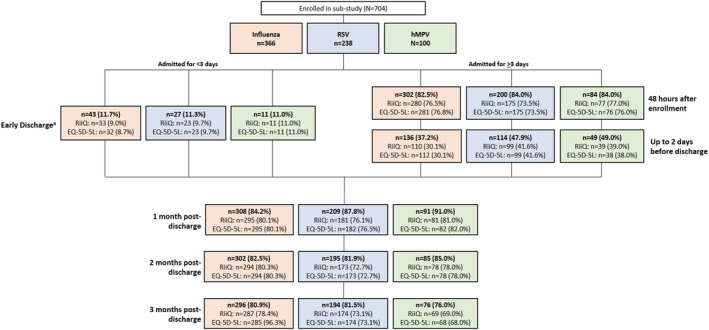
Respiratory Intensity and Impact Questionnaire (RiiQ™) and EQ‐5D‐5L responders at substudy assessment by time of assessment and pathogen. EQ‐5D‐5L: EuroQol 5 Dimensions 5 Levels; hMPV: Human Metapneumovirus; RiiQ™: Respiratory Intensity and Impact Questionnaire; RSV: Respiratory Syncytial Virus. This figure presents the number of responders (and % of total pathogen group enrolled in substudy) at each assessment timepoint for the patient‐reported outcomes of interest (RiiQ and EQ‐5D‐5L). ^a^If a participant was hospitalized for a short period (i.e., <72 h) or transferred to another ward, an early discharge assessment was performed on the day of discharge

This was a prospective cohort study in adult patients (≥18 years of age) hospitalized with ARTI at 40 centers across 12 countries (Australia, Argentina, Brazil, Canada, France, Germany, Japan, Malaysia, Mexico, Republic of Korea, South Africa, USA), over two consecutive influenza/RSV/hMPV epidemic seasons (2017–2019). The clinical diagnosis of ARTI and the decision to hospitalize the patient were made according to local standard of care (SOC) practices. The enrollment period followed the local respiratory viral infection seasonal progression. Participants were consented and enrolled in the main study within 24 h after admission. Viral testing was done as part of SOC or by study‐specific molecular diagnostic test if not performed as SOC. Participants with influenza (capped to 380 consecutive subjects), RSV, or hMPV infection confirmed by reverse transcription‐polymerase chain reaction were invited to enroll in the substudy, which comprised a hospitalization phase with 3 visits (enrollment, 48 h after enrollment/early discharge, and up to 2 days prior to discharge), and follow‐up telephone interviews at 1, 2, and 3 months post‐discharge (Figure 1). Additional details on study design have been presented elsewhere.^9^

### 2.2 Data collection

The RiiQ™ was developed from the FluiiQ™ and assesses lower respiratory tract (LRT), upper respiratory tract (URT), and systemic symptoms (Table 1).^1^, ^10^ Scores based on LRT and URT symptoms can be aggregated into an overall respiratory symptoms domain. Patients rate the 13 symptoms at their worst during the past 24 h on a four‐point (0 = none to 3 = severe) Likert scale. Domain scores are calculated by averaging LRT, URT, Systemic and all symptoms to create domain scores.

**TABLE 1:** FluiiQ™ and RiiQ™ symptoms domains and items

	Influenza intensity and impact questionnaire (FluiiQ™) items^a^	Respiratory intensity and impact questionnaire (RiiQ™) items^a^
Systemic symptoms domain	Feeling feverish	Feeling feverish
	Headache	Headache
	Neck pain	Neck pain
	Fatigue	Fatigue
	Loss of appetite	Loss of appetite
	Interrupted sleep	Interrupted sleep
	Body aches	Body aches
Upper respiratory tract (URT) domain	Sore throat	Sore throat
	Nasal congestion	Nasal congestion
Lower respiratory tract (LRT) domain	Cough	Cough
		Wheezing
		Expectoration
		Short of breath (SoB)

^a^ Full items available from the copyright holder at info@measuredsolutions.com.au.

The EQ‐5D‐5L measures health‐related quality of life (HRQoL) for cost‐effectiveness analysis based on five dimensions (Mobility, Self‐Care, Usual Activities, Pain/Discomfort, and Anxiety/Depression) rated on five levels ranging from “No Problems” to “Extreme Problems.”^11^ In addition, the EQ‐5D Visual Analog Scale (EQ‐VAS) records a participant's self‐rated health overall.^12^

Clinical information and PROs were collected during hospitalization and post‐discharge. The RiiQ™ Symptom Scale and EQ‐5D‐5L were administered as interviews during hospitalization (at 48 h after enrollment/early discharge and within 2 days before discharge) and follow‐up. If the participant was discharged within less than 72 h from the enrollment visit (early discharge), post‐baseline assessments were performed on the day of discharge, so the assessment within 2 days before discharge was not available. If the participant could not be interviewed, RiiQ™ Symptom Scale and EQ‐5D‐5L were not assessed.

### 2.3 Statistical analysis

The RiiQ™ symptoms domain scores were calculated by averaging the individual symptom severity scores. At each visit, RiiQ™ symptom scores were summarized overall, by pathogen, and by presence of core risk factors (CRF) (age ≥65 years, chronic obstructive pulmonary disease [COPD], asthma, chronic heart or chronic renal disease) for progression to severe disease and presence of long‐term sequalae and compared among groups using Kruskal–Wallis test. Individual RiiQ™ symptom scores were compared among pathogens and by presence/absence of CRF (as a binary variable, irrespective of the number of CRFs reported) using Fishers Exact Test to compare symptom severity. *P* values were not adjusted for multiple comparisons. The EQ‐5D‐5L Index Value was calculated by assigning a level code of 1–5 (“No Problems” to “Extreme Problems”) to each of the 5 dimensions, and the health state obtained was then mapped to a single index value by using the crosswalk value set available for the United Kingdom.^13^ Scatterplots with Spearman correlation coefficients compared RiiQ™ Symptom Scale scores with EQ‐VAS score and EQ‐5D‐5L index scores at 48 h after enrollment/early discharge and 1, 2, and 3 months post‐discharge.

## 3 RESULTS

Of the 3861 main study participants, 366 (9.5%) influenza‐positive, 238 (6.2%) RSV‐positive, and 100 (2.6%) hMPV‐positive participants were enrolled in the substudy. The mean (SD) age of substudy participants was 65.6 (16.2) years, with mean (SD) age in years higher among RSV participants (67.3 [16.5]) compared to influenza (64.4 [16.1], *p* = 0.032). Most (80.4%; *n* = 566) substudy participants presented with at least 1 CRF, although nearly 1 in 5 (19.6%; *n* = 138) had no CRF. A higher proportion of RSV participants than influenza patients had any CRF (86.1% versus 75.4%, respectively, *p* = 0.002). Age (≥65 years) was the most common CRF (influenza: 51.9%: RSV: 60.9%; hMPV: 57.0%), followed by heart disease (influenza: 38.8%; RSV: 41.6%; hMPV: 33.0%). Refer to Supplementary Materials for demographic and baseline clinical characteristics of this substudy population.

### 3.1 RiiQ™ Symptom Scale results

Overall, RiiQ™ Symptom Scale data were available for 667 of 704 substudy subjects, including 346 (94.5%) patients with confirmed influenza, 224 (94.1%) with RSV, and 97 (97.0%) with hMPV. RiiQ™ Symptom Scale data was available for 85.1% of participants at 48 h after enrollment/early discharge, 35.4% of participants at 2 days before discharge (for those hospitalized >3 days), and 79.3%, 77.4%, and 75.3% of participants at 1, 2, and 3 months post‐discharge, respectively (Figure 1).

### 3.2 Symptom domain score analysis

The mean (SE) LRT domain score was higher at 48 h after enrollment/early discharge (1.27 [0.03]) compared to Systemic and URT symptom domain scores (0.83 [0.03] and 0.65 [0.03], respectively). The mean (SE) LRT domain score decreased during follow‐up to 0.32 (0.02) at 3 months post‐discharge, yet remained higher than the mean (SE) Systemic (0.23 [0.02]) and URT (0.16 [0.02]) domain scores at 3 months post‐discharge (Table S2).

### 3.3 Symptom domain score analysis by pathogen

**FIGURE 2:**
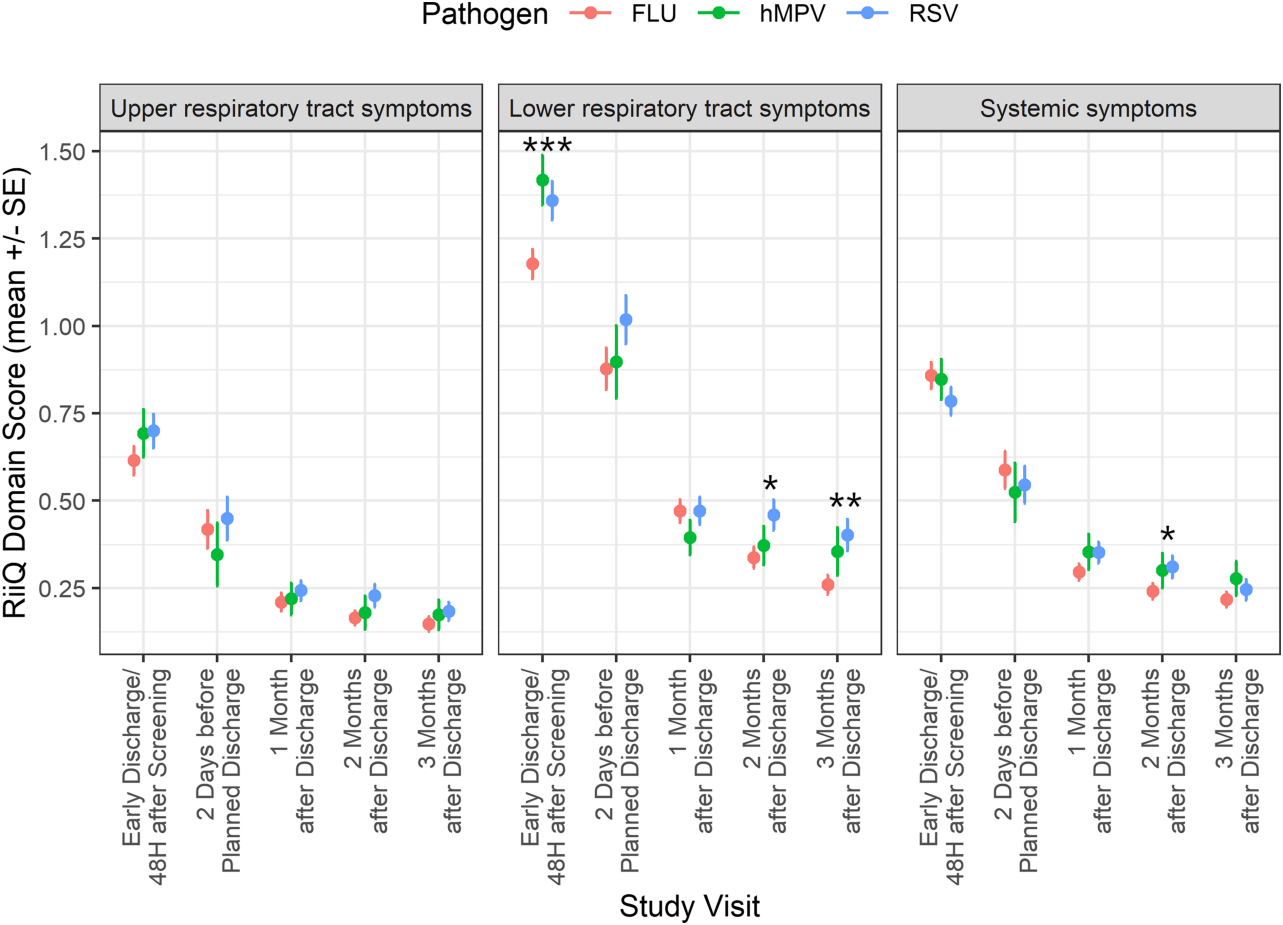
Respiratory Intensity and Impact Questionnaire (RiiQ™) domain scores over time by pathogen. FLU: Influenza; hMPV: Human Metapneumovirus; RiiQ™: Respiratory Intensity and Impact Questionnaire; RSV: Respiratory Syncytial Virus. Co‐infections (*N* = 5) were excluded. If a participant was hospitalized for a short period (i.e., <72 h) or transferred to another ward, an early discharge assessment was performed on the day of discharge. RiiQ™ data were available for 599 (85.0%) participants at 48 h after enrollment/early discharge, 249 (35.3%) participants at 2 days before discharge (for those hospitalized >3 days), and 558 (79.3%), 545 (77.4%), and 530 (75.3%) of the 704 participants with confirmed influenza, RSV or hPMV infection at 1, 2, and 3 months post‐discharge, respectively. *p* values: * *p* < 0.05; ** *p* < 0.01; *** *p* < 0.001 (RiiQ™ domain scores were compared among pathogens using Kruskal–Wallis test)

A significant difference sin mean (SE) LRT domain score was observed among the three pathogens at 48 h after enrollment/early discharge (influenza: 1.18 [0.04]; RSV: 1.36 [0.05]; hMPV: 1.42 [0.07], *p* = 0.001), 2 months post‐discharge (influenza: 0.34 [0.03]; RSV: 0.46 [0.04]; hMPV: 0.37 [0.06], *p* = 0.035), and 3 months post‐discharge (influenza: 0.26 [0.03]; RSV: 0.40 [0.04]; hMPV: 0.36 [0.07], *p* = 0.007). No other important differences were observed among the three pathogen groups for mean URT or systemic symptom scores during hospitalization or post‐discharge (Figure 2 and Table S2).

### 3.4 Symptom domain score analysis by core risk group

**FIGURE 3:**
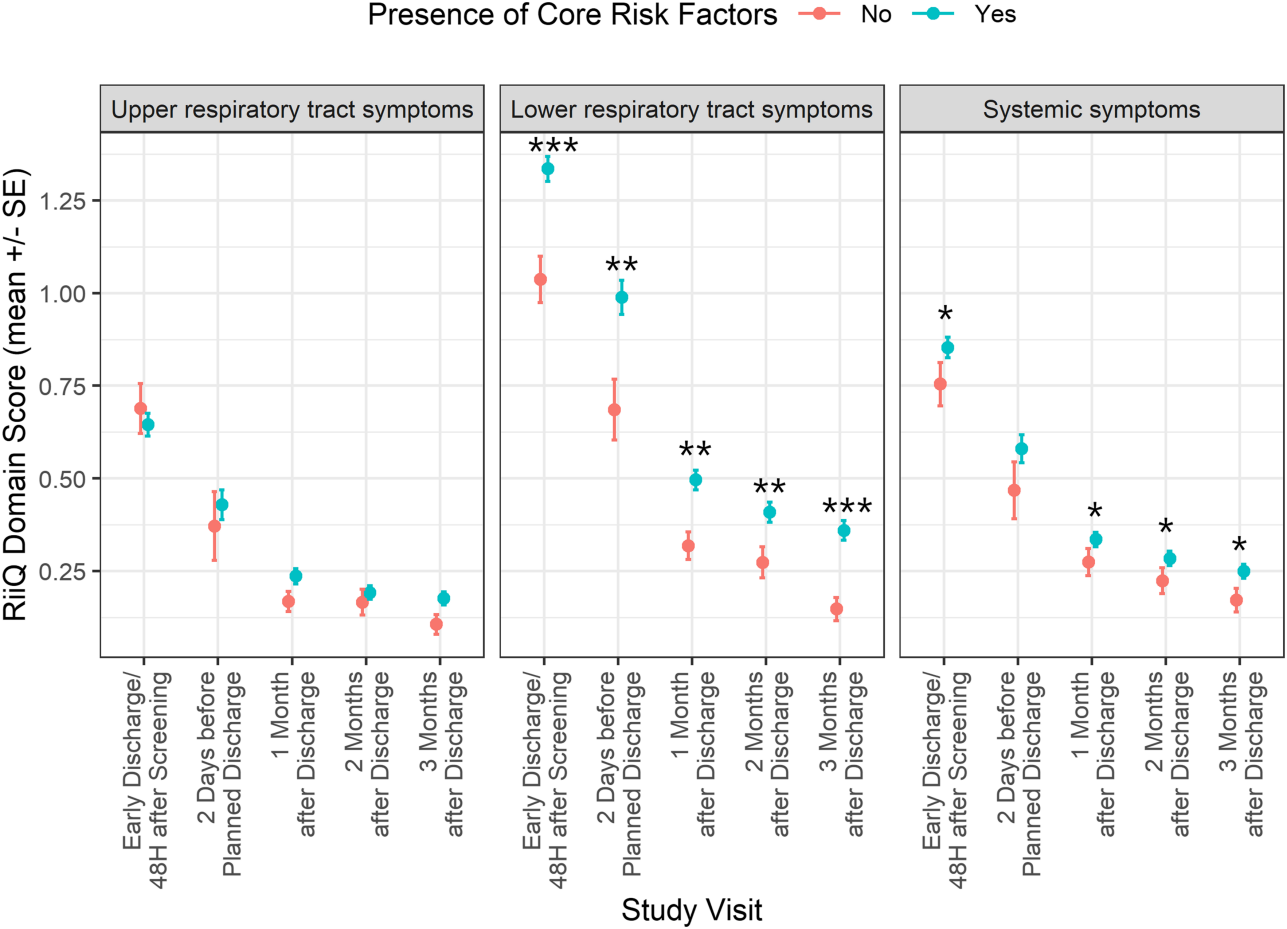
Respiratory Intensity and Impact Questionnaire (RiiQ™) domain scores over time by presence of core risk factors (CRF). SE: Standard error. If a participant was hospitalized for a short period (i.e., <72 h) or transferred to another ward, an early discharge assessment was performed on the day of discharge. Co‐infections (*N* = 5) were excluded. RiiQ™ data were available for 599 (85.0%) participants at 48 h after enrollment/early discharge, 249 (35.3%) participants at 2 days before discharge (for those hospitalized >3 days), and 558 (79.3%), 545 (77.4%), and 530 (75.3%) of the 704 participants with confirmed influenza, RSV or hPMV infection at 1, 2, and 3 months post‐discharge, respectively. *p* values: * *p* < 0.05; ** *p* < 0.01; *** *p* < 0.001 (RiiQ™ domain scores were compared among pathogens using Kruskal–Wallis test)

Mean (SE) LRT domain scores were higher for CRF(+) compared to CRF(−) participants at all time points during hospitalization and post‐discharge (48 h after enrollment/early discharge: 1.34 [0.03] versus 1.04 [0.06], *p* < 0.001; 2 days before discharge: 0.99 [0.05] versus 0.69 [0.08], *p* = 0.006; 1 month post‐discharge: 0.50 [0.03] versus 0.32 [0.04], *p* = 0.007; 2 months post‐discharge: 0.41 [0.03] versus 0.27 [0.04], *p* = 0.007; and 3 months post‐discharge: 0.36 [0.03] versus 0.15 [0.03], *p* < 0.001). Mean (SE) systemic symptom domain scores were higher for CRF(+) participants at each assessment: 48 h after enrollment/early discharge (0.85 [0.03] versus 0.75 [0.06], *p* = 0.034), 1 month post‐discharge (0.34 [0.02] versus 0.27 [0.03], *p* = 0.028), 2 months post‐discharge (0.28 [0.02] versus 0.22 [0.03], p = 0.046), and 3 months post‐discharge (0.25 [0.02] versus 0.17 [0.03], p = 0.015). No significant differences between CRF(+) and CRF(−) participants were observed for mean URT symptoms domain score (Figure 3 and Table S3).

### 3.5 Distribution of moderate‐to‐severe symptoms, based on individual symptoms score overall

**FIGURE 4:**
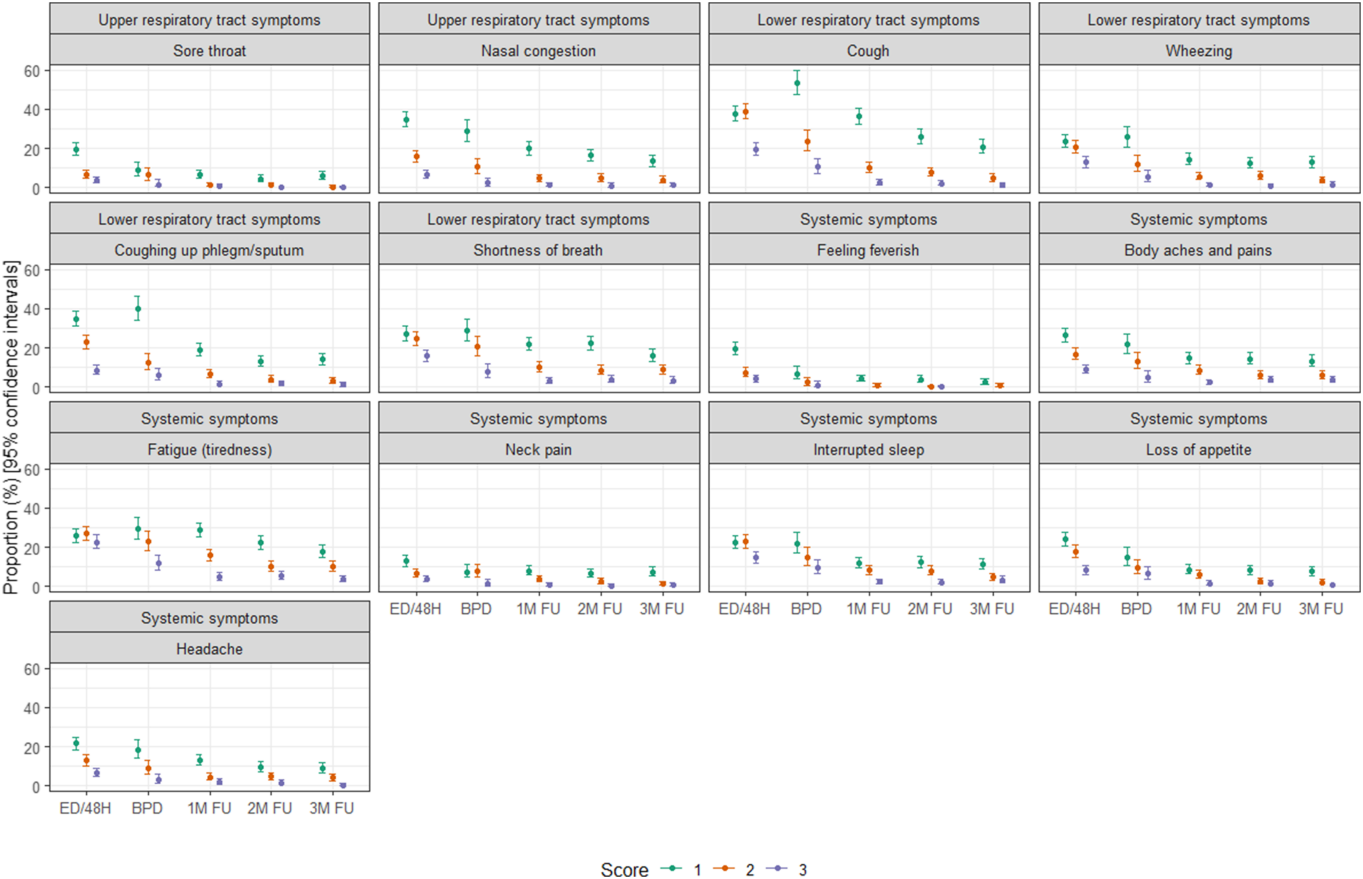
Frequency of Respiratory Intensity and Impact Questionnaire (RiiQ™) symptom score by visit. ED/48H: early discharge/48 h after screening; BPD: 2 days before planned discharge; 1 M FU: 1 month post‐discharge; 2 M FU: 2 months post discharge; 3 M FU: 3 months post discharge. This figure presents the frequency of 13 RiiQ Symptom Scores (range 1–3) at each visit, presented by domain. Scores of 0 are not shown in this figure. RiiQ™ data were available for 599 (85.0%) participants at 48 h after enrollment/early discharge, 249 (35.3%) participants at 2 days before discharge (for those hospitalized >3 days), and 558 (79.3%), 545 (77.4%), and 530 (75.3%) of the 704 participants with confirmed influenza, RSV or hPMV infection at 1, 2, and 3 months post‐discharge, respectively

At the first assessment (48 h after enrollment/early discharge), the most common symptoms with moderate‐to‐severe level (score of 2 or 3) were cough (58.6%), fatigue (49.6%), SOB (40.4%), interrupted sleep (37.3%), and wheezing (33.7%). The proportion of participants with moderate‐to‐severe symptoms decreased during follow‐up regardless of the pathogen, though several moderate‐to‐severe symptoms were present in >10% participants at 2 months post‐discharge (fatigue: 15.6%; SOB: 12.5%; interrupted sleep: 10.3%) and at 3 months post‐discharge (fatigue: 13.6%; SOB: 12.3%) (Figure 4).

### 3.6 Distribution of moderate‐to‐severe symptoms, based on individual symptoms score by pathogen

A statistically significant difference in frequency of moderate‐to‐severe LRT symptoms was observed among the pathogen groups at 48 h after enrollment/early discharge for SOB (influenza: 34.5%; RSV: 45.5%; hMPV: 50.0%, *p* < 0.01) and wheezing (influenza: 28.1%; RSV: 35.9%; hMPV: 48.9%, *p* < 0.01) and at 3 months post‐discharge for SOB (influenza: 9.1%; RSV: 16.1%; hMPV: 15.9%, *p* < 0.05) and cough (influenza: 3.5%; RSV: 8.6%; hMPV: 10.1%, *p* < 0.05). When excluding hMPV participants, RSV participants also reported a greater frequency of moderate‐to‐severe SOB at 1 month post‐discharge (influenza: 10.8%; RSV: 18.2%, *p* < 0.05) compared with influenza participants.

**FIGURE 5:**
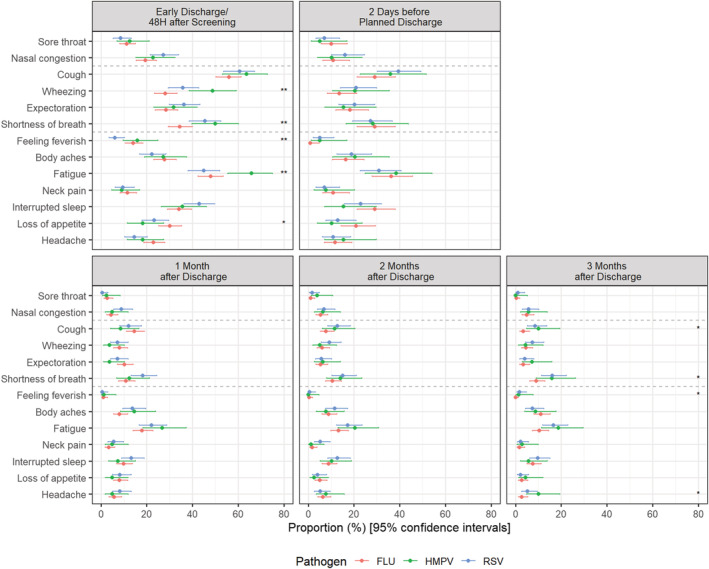
Frequency of participants with moderate‐to‐severe symptoms by pathogen and visit. FLU: Influenza; hMPV: Human metapneumovirus; RSV: Respiratory syncytial virus. 95% Confidence intervals calculated using Wilson's method. p‐values based on Fisher‐exact test: * *p* < 0.05; ** *p* < 0.01; *** *p* < 0.001. If a participant was hospitalized for a short period (i.e., <72 h) or transferred to another ward, an early discharge assessment was performed on the day of discharge. RiiQ™ data were available for 599 (85.0%) participants at 48 h after enrollment/early discharge, 249 (35.3%) participants at 2 days before discharge (for those hospitalized >3 days), and 558 (79.3%), 545 (77.4%), and 530 (75.3%) of the 704 participants with confirmed influenza, RSV or hMPV infection at 1, 2, and 3 months post‐discharge, respectively

A statistically significant difference in frequency of moderate‐to‐severe systemic symptoms was observed at 48 h after enrollment/early discharge for fatigue (influenza: 47.9%; RSV: 44.9%; hMPV: 65.9%, *p* < 0.01) and feeling feverish (influenza: 14.1%; RSV: 6.1%; hMPV: 15.9%, *p* < 0.01). When excluding hMPV participants, at 48 h after enrollment/early discharge, influenza participants reported a greater frequency of moderate‐to‐severe headache (influenza: 23.0%, RSV:14.6%, *p* < 0.05), while RSV participants reported a greater frequency of moderate‐to‐severe interrupted sleep (influenza: 34.2%; RSV: 42.9%, *p* < 0.05) (Figures 5 and S1).

### 3.7 Distribution of moderate‐to‐severe symptoms, based on individual symptoms score by core risk group

A greater proportion of CRF(+) participants (*n* = 535) versus CRF(−) participants (*n* = 132) reported moderate‐to‐severe LRT symptoms, including wheezing, at all assessments (48 h after enrollment/early discharge: 36.4% versus 23.6%, *p* < 0.01; 2 days before discharge: 20.4% versus 4.7%, *p* < 0.05; 1 month post‐discharge: 8.6% versus 1.7%, *p* < 0.01; 2 months post‐discharge: 8.4% versus 1.7%, *p* < 0.05; 3 months post‐discharge: 6.6% versus 1.0%, *p* < 0.05), as well as moderate‐to‐severe SOB at 48 h after enrollment/early discharge (45.1% versus 22.8%, *p* < 0.001), 1 month post‐discharge (16.1% versus 3.5%, p < 0.001), and 3 months post‐discharge (13.8% versus 5.8%, p < 0.05). A greater proportion of CRF(+) participants also reported moderate‐to‐severe cough at (60.8 versus 50.4%, *p* < 0.05) at 48 h after enrollment/early discharge compared to CRF(−) participants.

Considering the incidence of systemic symptoms, a greater proportion of CRF(+) participants versus CRF(−) participants reported moderate‐to‐severe fatigue (21.7% versus 16.4%, *p* < 0.01) at 48 h after enrollment/early discharge, loss of appetite (5.3% versus 0.9%, *p* < 0.05) at 2 months post‐discharge, and body aches and pains (11.0% versus 3.9%, *p* < 0.05) at 3 months post‐discharge.

**FIGURE 6:**
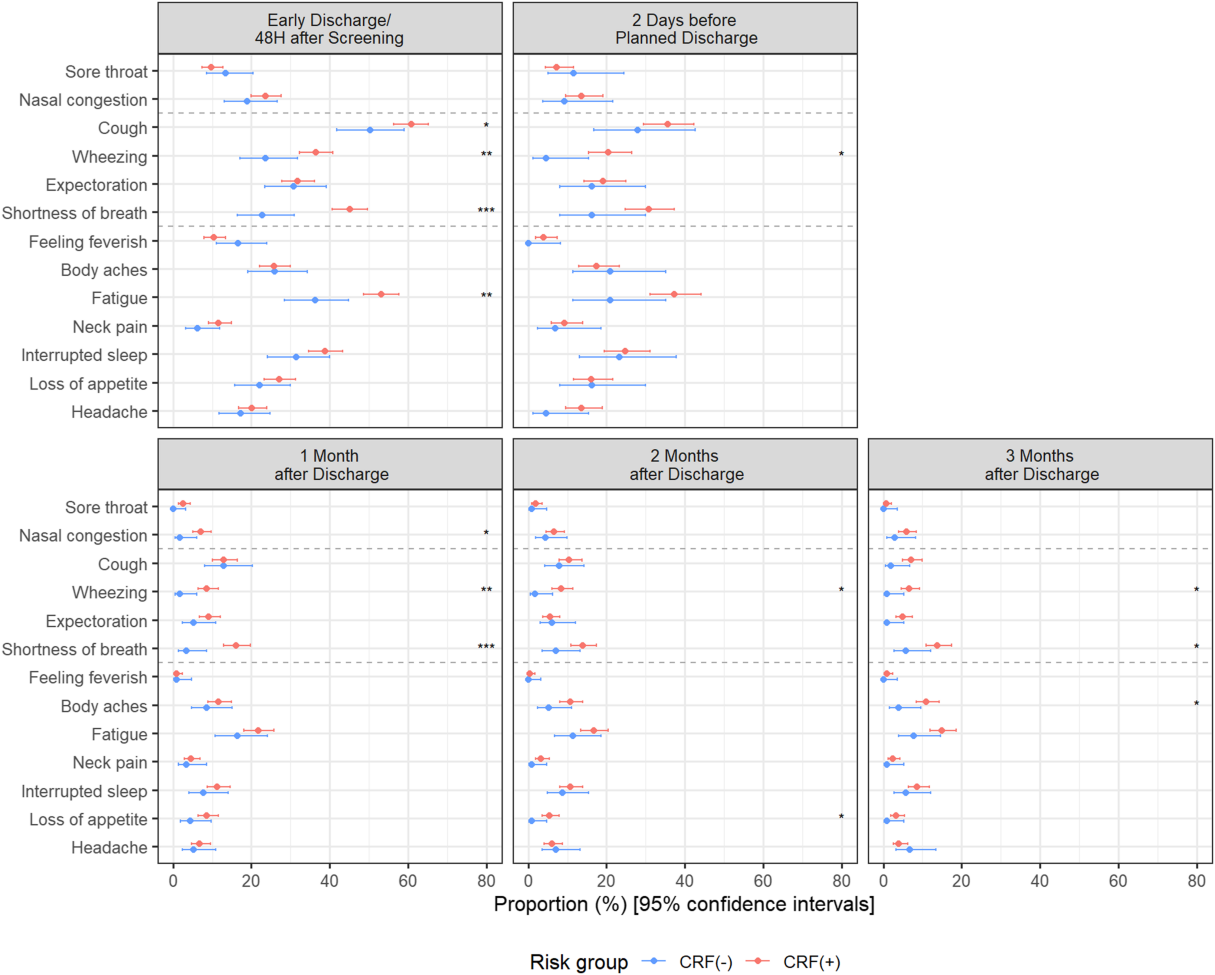
Frequency of participants with moderate‐to‐severe symptoms by presence of CRF and visit. CRF: Core risk factors (age ≥65, chronic heart or renal disease, chronic obstructive pulmonary disease [COPD], asthma). 95% Confidence intervals calculated using Wilson's method. *p* values based on Fisher‐exact test: * *p* < 0.05; ** *p* < 0.01; *** *p* < 0.001. If a participant was hospitalized for a short period (i.e., <72 h) or transferred to another ward, an early discharge assessment was performed on the day of discharge. RiiQ™ data were available for 599 (85.0%) participants at 48 h after enrollment/early discharge, 249 (35.3%) participants at 2 days before discharge (for those hospitalized >3 days), and 558 (79.3%), 545 (77.4%), and 530 (75.3%) of the 704 participants with confirmed influenza, RSV or hPMV infection at 1, 2, and 3 months post‐discharge, respectively

Overall, some moderate‐to‐severe systemic and LRT symptoms Moderate‐to‐severe fatigue and SOB persisted in >10% of participants, including fatigue and SOB at 2 months post‐discharge (15.6% and 12.5%, respectively) and 3 months post‐discharge (13.6% and 12.3%, respectively) (Figure 6).

### 3.8 Association between RiiQ™ symptom scores and EQ‐5D‐5L

Among 704 participants in the substudy, EQ‐VAS score was available for 84.2% of participants at 48 h after enrollment/early discharge, 35.4% of participants at 2 days before discharge, and 79.0%, 77.4%, and 75.0% of participants at 1, 2, and 3 months post‐discharge, respectively. EQ‐5D‐5L Index Value score was assessed for 84.9% of participants at 48 h after enrollment/early discharge, 35.4% of participants at 2 days before discharge, and 79.4%, 77.4%, and 74.9% of participants at 1, 2, and 3 months post‐discharge, respectively (Figure 1 *)*.

**FIGURE 7:**
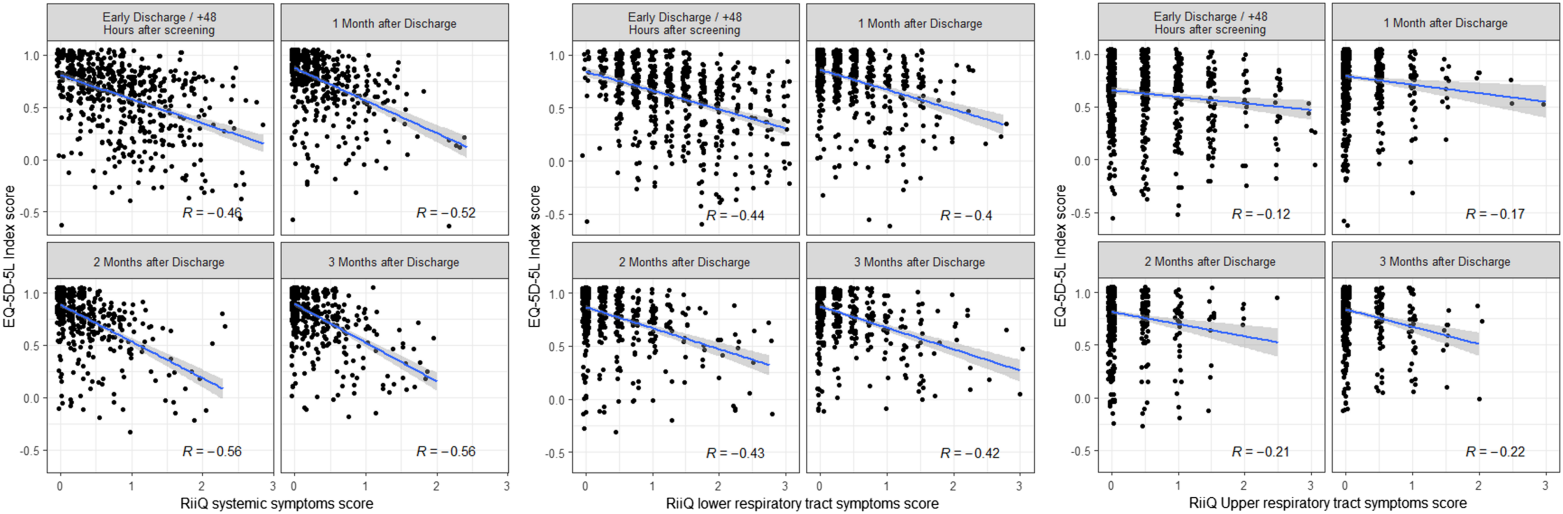
Association between Respiratory Intensity and Impact Questionnaire (RiiQ™) score and EQ‐5D‐5L index value score over time. EQ‐5D‐5L = EuroQol 5 Dimensions 5 Levels; RiiQ™ = Respiratory Intensity and Impact Questionnaire. Small random jitter is applied to the data points to allow for a clearer view on the data points. Spearman correlation coefficient (*R*) is presented. A linear regression line (with 95% confidence interval) is added to aid visualization. Co‐infections (*N* = 5) were excluded. EQ‐5D‐5L Index Value Scores <0 indicate a health state worse than death (likely due to underlying conditions)

Both EQ‐VAS and EQ‐5D‐5L Index Value scores were compared with RiiQ™ Symptom Scores. EQ‐VAS score was moderately and negatively associated with RiiQ™ LRT (*R* ranging from −0.40 to −0.43) and Systemic symptom domain scores (*R* ranging from −0.41 to −0.50). EQ‐5D‐5L Index Value score was also moderately and negatively associated with LRT (*R* ranging from −0.40 to −0.44) and Systemic symptom domain scores (*R* ranging from −0.46 to −0.56). RiiQ™ URT domain score showed low association with EQ‐VAS score (*R* ranging from −0.16 to −0.29) and EQ‐5D‐5L Index Value score (*R* ranging from −0.12 to −0.22) (Figures 7 and S4).

## 4 DISCUSSION

The HARTI substudy aimed to describe the disease burden in adults hospitalized with influenza, RSV, and hMPV infection during hospitalization and up to 3 months post‐discharge. The novel RiiQ™ Symptom Scale was used to describe participants' self‐rated symptom severity. RiiQ™ Symptom Scale scores demonstrated significantly increased symptom severity for participants with either RSV or hMPV infection compared to those with influenza. This was particularly driven by the LRT domain. At 48 h after enrollment/early discharge, a significantly greater frequency of moderate‐to‐severe wheezing and SOB for RSV and hMPV compared to influenza was observed. Additionally, at 3 months post‐discharge, significant differences persisted with more moderate‐to‐severe SOB and cough observed for RSV and hMPV infected patients. These results suggest that wheezing is an important component of the patient‐reported symptoms related to RSV, hMPV and influenza. Compared with influenza, more severe LRT symptoms were reported for RSV and hMPV participants during hospitalization. Furthermore, while all three pathogen groups may experience a similar recovery initially post‐discharge, those with either RSV or hMPV infection may report more long‐lasting LRT symptoms compared to participants after influenza infection. Though, this may have been confounded by a greater proportion of RSV and hMPV patients reporting underlying COPD or asthma. Demographics and underlying conditions differed across pathogen groups; those with either RSV or hMPV infection were older (RSV: *p* = 0.032; hMPV: *p* = 0.437) and were more likely to have risk factors for severe illness (RSV: *p* = 0.002; hMPV: *p* = 0.058) compared to those in the influenza group (Table S1).

The HARTI study suggests that hospitalized RSV patients experience a significantly greater burden of LRT symptoms, including cough, wheezing, and SOB, during hospitalization and post‐discharge. A recent study in the sentinel influenza surveillance system in Portugal, investigating signs and symptoms as clinical predictors for RSV infection, determined that the European Union (EU) influenza‐like case definition of at least one respiratory symptom (cough, sore throat, or SOB) and at least one systemic symptom (fever, malaise, headache, or myalgia) was not accurate for RSV detection. The two other case definitions evaluated (a modified EU acute respiratory infection [sudden onset of symptoms including at least one among: cough, sore throat or SOB] and one respiratory symptom [at least one among: cough, sore throat or SOB]) were not able either to sufficiently discriminate RSV‐positive cases, indicating the need for a more sensitive case definition to avoid the underestimation of RSV disease burden.^14^ However, the ILI and ARI case definitions are broad and unspecific, even for influenza detection. The results of the HARTI study are consistent with these findings, demonstrated that there was a general overlap of symptoms reported for RSV, hMPV, and influenza infection, with some distinctive features to warrant new tools to accurately assess illness. While case definitions typically have only symptom present versus absent to identify an ARTI, the RiiQ™ offers the ability to quantify disease severity given the comprehensive range of symptoms and use of a four‐point grading (none to severe) of symptoms.

The RiiQ™ Symptom Scale scores also demonstrated increased symptom severity for CRF(+) participants compared to CRF(−) participants, which was particularly driven by LRT and Systemic Symptom Scale scores. A majority of hospitalized participants in the HARTI study presented with at least one CRF (80.4%). During hospitalization, CRF(+) participants reported significantly greater frequencies of moderate‐to‐severe LRT symptoms, including cough, wheezing, and SOB, as well as moderate‐to‐severe systemic symptom of fatigue. At 3 months post‐discharge, CRF(+) participants reported significantly greater frequencies of moderate‐to‐severe LRT symptoms, including wheezing, SOB, and expectoration, as well as significantly greater frequencies of systemic symptoms, including fatigue and body aches, compared to CRF(−) participants. It suggests that presence of CRF is associated with more severe LRT engagement and aligns with the expectation that CRF(+) patients may have significantly greater disease burden and longer‐lasting symptoms compared to CRF(−) participants.

For new PRO questionnaires, comparison with other widely used questionnaires, such as EQ‐5D‐5L, helps to understand measurement properties, building confidence that the new tool does perform as expected and that the type and severity of symptoms monitored are relevant for the HRQoL of the patient.^15^ The HARTI study evidenced a moderate (>0.30) association between RiiQ™ and EQ‐5D‐5L Index Value at all time points for Systemic (min: −0.46, max: −0.56) and LRT domains (min: −0.40, max: −0.44), while low association was observed for URT domain scores. Similarly, a moderate association was observed between RiiQ™ and EQ‐VAS at all time points for Systemic (min: −0.41, max: −0.50) and LRT domains (min: −0.40, max: −0.44). The EQ‐5D‐5L Index Value and EQ‐VAS have previously been shown to be associated with other disease‐specific questionnaires, including the Chronic Respiratory Disease Questionnaire.^16^ LRT and systemic symptoms, domains as measured using RiiQ™, appear to be moderately associated with poor health status, as measured using EQ‐5D‐5L.

A limitation of this study is that the first PRO assessment was administered 48 h after the pathogen was identified and the enrollment of the patient in the substudy was confirmed. The frequency of moderate‐to‐severe symptoms may have been greater at hospital admission; future studies may explore whether a greater difference would be observed among the three pathogen groups at the time of pathogen diagnosis, when symptoms may be most severe. Assessments performed at 48 h after enrollment were pooled with those performed at early discharge (roughly 10% of the analysis set); inclusion of early discharge patients (<3 days) may have resulted in less severe disease observed at this timepoint. By study design, having an evaluation 48 h after enrollment, the second one 48 h before discharge would be performed only for patients with longer hospitalization. The “within 2 days before discharge” time point had considerable missing data, due to the challenges of operational implementation of the last assessment prior to discharge; however, the compliance with PRO data collection was high for the time points before and after the hospital discharge. The pre‐infection symptoms were not available; therefore, one cannot distinguish between the long‐term respiratory disease sequela and symptoms related to the underlying chronic condition for the CRF(+) group (e.g., age ≥65 years, COPD, asthma).

Overall, results from the HARTI study allow for comparative assessment of symptoms between the 3 most common viral respiratory pathogens and provide novel evidence regarding symptom severity and HRQoL burden for influenza, RSV, and hMPV. The RiiQ™ is currently being used in clinical development for RSV vaccine and antiviral studies, and the data presented in this manuscript support continued use of the RiiQ™ for characterization of these patients.^8^

## 5 CONCLUSION

The majority of hospitalized influenza, RSV and hMPV patients were older than 65 years, with the RSV group slightly older compared with influenza group. RiiQ™ showed greater incidence of moderate‐to‐severe LRT symptoms for RSV and hMPV compared with influenza. Additionally, presence of CRF was associated with greater moderate‐to‐severe symptom burden during hospitalization and post‐discharge compared to those without CRF, with significantly higher LRT and systemic symptom domain scores at 1, 2, and 3 months post‐discharge. The association between RiiQ™ Symptom Scale and EQ‐5D‐5L is encouraging and warrants further exploration of RiiQ™ for pharmaceuticals development.

## AUTHOR CONTRIBUTIONS

**Ann Falsey:** Investigation; methodology. **Edward Walsh:** Investigation; methodology. **Richard Osborne:** Methodology. **Yannick Vandendijck:** Formal analysis; methodology. **Xiaohui Ren:** Project administration. **James Witek:** Conceptualization; funding acquisition; methodology; supervision. **Diye Kang:** Formal analysis. **Eric Chan:** Methodology. **Jane Scott:** Conceptualization; methodology. **Gabriela Ispas:** Conceptualization; formal analysis; methodology; project administration.

## CONFLICTS OF INTEREST

*Potential conflict of interest*. A. Falsey has received research grants from Pfizer, Mark Sharp and Dohme, Janssen, and BioFire, and is on the Data Safety and Monitoring Board for Novavax.

*Potential conflict of interest*. J. Witek. J. Scott, and *E. Chan* are J&J stockholders.

*Potential conflict of interest*. Y. Vandenijck, X. Ren, D. Kang, *E. Chan* and G. Ispas are employees of Janssen Pharmaceutica NV.

*Potential conflict of interest*. E. Walsh has received grants from Merck, Janssen, Pfizer, and was a paid member Data Safety and Monitoring Board for GSK.

*Potential conflict of interest*. R. Osborne RO has provided research services to Measure Solutions for Health P/L, Janssen, Merck and GSK.

### PEER REVIEW

The peer review history for this article is available at https://publons.com/publon/10.1111/irv.12903.

## Supporting information


**Data S1.** Supporting Information

## Data Availability

The data that support the findings of this study are available on request from the corresponding author. The data are not publicly available due to privacy or ethical restrictions.
