# Supplementary material for: Comparative assessment of reported symptoms of influenza, respiratory syncytial virus, and human metapneumovirus infection during hospitalization and post‐discharge assessed by Respiratory Intensity and Impact Questionnaire
Source: Influenza Other Respir Viruses. 2021 Sep 2;16(1):79–89. doi: 10.1111/irv.12903 (PMC8692817; doi:10.1111/irv.12903)
Supplement: Supplementary file 1 — Data S1. Supporting Information [file IRV-16-79-s001.docx]

**Title:** Clinical Burden of Influenza, Respiratory Syncytial Virus, and Human Metapneumovirus Infection During Hospitalization and Post-discharge Assessed by Respiratory Intensity and Impact Questionnaire (RiiQ^TM^) [Supplementary Materials]

**Short running title:** HARTI RiiQ^TM^ Study

**Authors and Affiliations**

Ann R. Falsey^1^, Edward E. Walsh^1^, Richard H. Osborne^2^, Yannick Vandenijck^3^, Xiaohui Ren^3^, James Witek^4^, Diye Kang^3^, Eric Chan^5^, Jane Scott^5^, Gabriela Ispas^3^

1. *University of Rochester School of Medicine Rochester Regional Health, Rochester, NY, United States*
2. *Swinburne University of Technology, Australia*
3. *Janssen Infectious Diseases, Beerse, Belgium*
4. *Janssen Research & Development, LLC, Titusville, NJ, United States*
5. *Janssen Global Services, LLC, Raritan, NJ, United States*

**Funding:** This work was supported by Janssen Pharmaceutica NV.

**Acknowledgment:** HARTI Study Group (Ting Soo Chow, Xavier Duval, Thomas Harrer, Stacey House, Nobuhisa Ishikawa, Odile Launay, Jacob Lee, Analia Mykietiuk, Mozar Neto, Marina Okoshi, Jan Rupp, Imitar Sajkov, Desmind Samuel, Masaharu Shinkai, Heidi Siebert, Selim Suner, Seong-Heon Wie, IQVIA Real World Solutions [Pascale Peeters, Rupali Naik, Sara Waugh]), critical reading of the manuscript: Magda Opsomer and Roman Fleischhackl (Janssen)

**SUPPLEMENTARY TABLES AND FIGURES**

[Figure 1 Frequency of Influenza vs. RSV Participants With Moderate to Severe Symptoms by Visit 6](#_Toc69461485)

[Figure 2 EQ-5D-5L VAS Score over Time by Pathogen 11](#_Toc69461486)

[Figure 3 EQ-5D-5L VAS Score over Time by Presence of Core Risk Factors 12](#_Toc69461487)

[Figure 4 Association Between RiiQ^TM^ Score and EQ-5D-5L VAS Score Over Time 13](#_Toc69461488)

[Table 1 Demographic and Baseline Clinical Characteristics by Respiratory Pathogen 3](#_Toc72246755)

[Table 2 Mean RiiQ^TM^ Domain Score by Pathogen 7](#_Toc72246756)

[Table 3 Mean RiiQ^TM^ Domain Score by Presence of Core Risk Factor (CRF) 9](#_Toc72246757)

**Table 1 Demographic and Baseline Clinical Characteristics by Respiratory Pathogen**

|  | Influenza  (n=366) | RSV  (n=238) | hMPV  (n=100) | Total^a^  (n=709) |
| --- | --- | --- | --- | --- |
| Age (years) |  |  |  |  |
| Mean (SD) | 64.4 (16.05) | 67.3 (16.52) | 65.9 (15.65) | 65.6 (16.19) |
| Median | 65.5 | 70.0 | 69.0 | 67.0 |
| (Range) | (18; 99) | (18; 98) | (24; 93) | (18; 99) |
| P-value |  | 0.032^b^ | 0.437^c^ | 0.098^d^ |
| Female gender, n (%) | 193 (52.7%) | 147 (61.8%) | 62 (62.0%) | 404 (57.0%) |
| P-value |  | 0.035^b^ | 0.124^c^ | 0.051^d^ |
| Presence of core risk factor, n (%)^e^ | 276 (75.4%) | 205 (86.1%) | 85 (85.0%) | 570 (80.4%) |
| P-value |  | 0.002^b^ | 0.058^c^ | 0.002^d^ |
| Presence of core risk factor, n (%)^e^ | 276 (75.4%) | 205 (86.1%) | 85 (85.0%) | 570 (80.4%) |
| P-value |  | 0.002^b^ | 0.058^c^ | 0.002^d^ |
| Age ≥65 years | 190 (51.9%) | 145 (60.9%) | 57 (57.0%) | 395 (55.7%) |
| P-value |  | 0.036^b^ | 0.429^c^ | 0.089^d^ |
| Chronic disease-heart disease | 142 (38.8%) | 99 (41.6%) | 33 (33.0%) | 276 (38.9%) |
| P-value |  | 0.548^b^ | 0.345^c^ | 0.334^d^ |
| Chronic disease-renal disease | 46 (12.6%) | 39 (16.4%) | 22 (22.0%) | 109 (15.4%) |
| P-value |  | 0.231^b^ | 0.027^c^ | 0.055^d^ |
| Asthma | 47 (12.8%) | 51 (21.4%) | 20 (20.0%) | 120 (16.9%) |
| P-value |  | 0.007^b^ | 0.099^c^ | 0.014^d^ |
| Respiratory tract morbidity-COPD | 81 (22.1%) | 73 (30.7%) | 32 (32.0%) | 187 (26.4%) |
| P-value |  | 0.024^b^ | 0.056^c^ | 0.026 ^d^ |
| Presence of other risk factor, n (%)^f^ | 57 (15.6%) | 28 (11.8%) | 11 (11.0%) | 96 (13.5%) |
| P-value |  | 0.232^b^ | 0.323^c^ | 0.292^d^ |
| COPD severity, n (%) |  |  |  |  |
| N | 81 | 73 | 32 | 187 |
| Mild | 20 (24.7%) | 10 (13.7%) | 6 (18.8%) | 36 (19.3%) |
| Moderate | 18 (22.2%) | 20 (27.4%) | 7 (21.9%) | 46 (24.6%) |
| Severe | 7 (8.6%) | 17 (23.3%) | 6 (18.8%) | 30 (16.0%) |
| Not reported | 36 (44.4%) | 26 (35.6%) | 13 (40.6%) | 75 (40.1%) |
| P-value |  | 0.032^b^ | 0.481^c^ | 0.1776^d^ |
| Previous vaccinations, n (%) |  |  |  |  |
| Influenza | 169 (46.2%) | 143 (60.1%) | 73 (73.0%) | 388 (54.7%) |
| P-value |  | 0.001^b^ | <0.001^c^ | <0.001^d^ |
| Pneumococcal | 132 (36.2%) | 93 (39.1%) | 58 (58.0%) | 286 (40.4%) |
| Missing | 1 (0.3%) | 0 | 0 | 1 (0.1%) |
| P-value |  | 0.508^b^ | <0.001^c^ | <0.001^d^ |
| Symptom length before hospitalization (days) |  |  |  |  |
| Mean (SD) | 4.5 (4.01) | 5.6 (7.27) | 5.1 (5.35) | 5.0 (5.50) |
| Median | 3.0 | 4.0 | 4.0 | 4.0 |
| Range | (0; 31) | (0; 92) | (0; 35) | (0; 92) |
| P-value |  | 0.013^b^ | 0.420^c^ | 0.045^d^ |
| Reason for hospital admission |  |  |  |  |
| Only ARTI | 165 (45.1%) | 100 (42.0%) | 51 (51.0%) | 319 (45.0%) |
| Only underlying medical conditions other than ARTI | 10 (2.7%) | 6 (2.5%) | 3 (3.0%) | 19 (2.7%) |
| Both ARTI and underlying medical conditions other than ARTI | 191 (52.2%) | 132 (55.5%) | 46 (46.0%) | 371 (52.3%) |
| P-value |  | 0.733^b^ | 0.548^c^ | 0.636^d^ |
| Type of ARTI, n(%) |  |  |  |  |
| Asthma exacerbation | 19 (5.3%) | 29 (12.5%) | 10 (10.3%) | 58 (8.4%) |
| P-value |  | 0.003^b^ | 0.124^c^ | 0.007^d^ |
| Bronchitis | 43 (12.1%) | 26 (11.2%) | 8 (8.2%) | 78 (11.3%) |
| P-value |  | 0.849^b^ | 0.380^c^ | 0.571^d^ |
| COPD exacerbation | 52 (14.6%) | 61 (26.3%) | 26 (26.8%) | 140 (20.3%) |
| P-value |  | 0.001 ^b^ | 0.008^c^ | 0.001^d^ |
| Other | 148 (41.6%) | 65 (28.0%) | 22 (22.7%) | 237 (34.3%) |
| P-value |  | 0.001 ^b^ | 0.001^c^ | 0.001^d^ |
| Pneumonia | 138 (38.8%) | 86 (37.1%) | 42 (43.3%) | 268 (38.8%) |
| P-value |  | 0.744 ^b^ | 0.489^c^ | 0.572^d^ |
| Missing | 10 (2.7%) | 6 (2.5%) | 3 (3.0%) | 19 (2.7%) |
| Type of underlying medical condition other than ARTI, n (%) |  |  |  |  |
| Asthma or COPD | 48 (23.9%) | 49 (35.5%) | 18 (36.7%) | 116 (29.7%) |
| Congestive heart failure | 24 (11.9%) | 21 (15.2%) | 5 (10.2%) | 50 (12.8%) |
| Sepsis | 26 (12.9%) | 13 (9.4%) | 4 (8.2%) | 43 (11.0%) |
| Hypoxemia | 53 (26.4%) | 39 (28.3%) | 12 (24.5%) | 105 (26.9%) |
| Other | 51 (24.9%) | 16 (11.6%) | 10 (20.4%) | 76 (19.5%) |
| O2 supplement at screening visit | 186 (50.8%) | 157 (66.0%) | 64 (64.0%) | 409 (57.7%) |
| P-value |  | <0.001^b^ | 0.026^c^ | <0.001^d^ |
| NEWS score at screening |  |  |  |  |
| Mean (SD) | 3.99 (2.723) | 4.64 (2.677) | 4.12 (2.461) | 4.22 (2.682) |
| Median | 4.00 | 5.00 | 4.00 | 4.00 |
| Range | (0.0; 13.0) | (0.0; 12.0) | (0.0; 11.0) | (0.0; 13.0) |
| Missing | 35 (9.6%) | 23 (9.7%) | 15 (15.0%) | 74 (10.4%) |
| P-value |  | 0.012^b^ | 0.589^c^ | 0.039^d^ |

ARTI: Acute Respiratory Tract Infection; COPD: Chronic Obstructive Pulmonary Disease; hMPV: Human Metapneumovirus; RSV: Respiratory Syncytial Virus; SD: Standard Deviation.

^a^ Participants with co-infections (between influenza and/or RSV and/or hMPV) (n=5) are not displayed in this table but are included in the Total column.

^b^ p-value based on Student t-test (age), Wilcoxon rank sum test (symptom length, NEWS score)) or chi-square tests (categorical variables) for the pairwise comparison between RSV and influenza participants.

^c^ p-value based on Student t-test (age), Wilcoxon rank sum test (symptom length, NEWS score) or chi-square tests (categorical variables) for the pairwise comparison between hMPV and influenza participants.

^d^ p-value based on one-way Anova (age), Kruskal-Wallis rank sum test (symptom length, NEWS score) or chi-square tests (categorical variables) testing for differences between Influenza, RSV and hMPV participants.

^e^ Core risk factors: Age ≥65, Chronic -heart disease, COPD, Chronic renal disease, Asthma.

^f^ Comorbidities: Chronic disease-heart disease, Chronic disease-HIV infection, Chronic disease-liver disease, Chronic disease-lung disease, Chronic disease-other, Chronic disease-renal disease, Congenital or acquired immunodeficiencies, Diabetes, Diagnosed atopy-asthma, Diagnosed atopy-hay fever, Diagnosed atopy-other, Neoplasia, Neurological and/or neuropsychiatric condition, Neuromuscular disorder-multiple sclerosis, Neuromuscular disorder-myasthenia gravis, Neuromuscular disorder-other, Other, Respiratory tract morbidity-COPD, Respiratory tract morbidity-cystic fibrosis, Respiratory tract morbidity-other, Respiratory tract morbidity-pulmonary hypertension, Upper airway abnormality-other, Upper airway abnormality-subglottic stenosis

**Figure 1 Frequency of Influenza vs. RSV Participants With Moderate to Severe Symptoms by Visit**

*
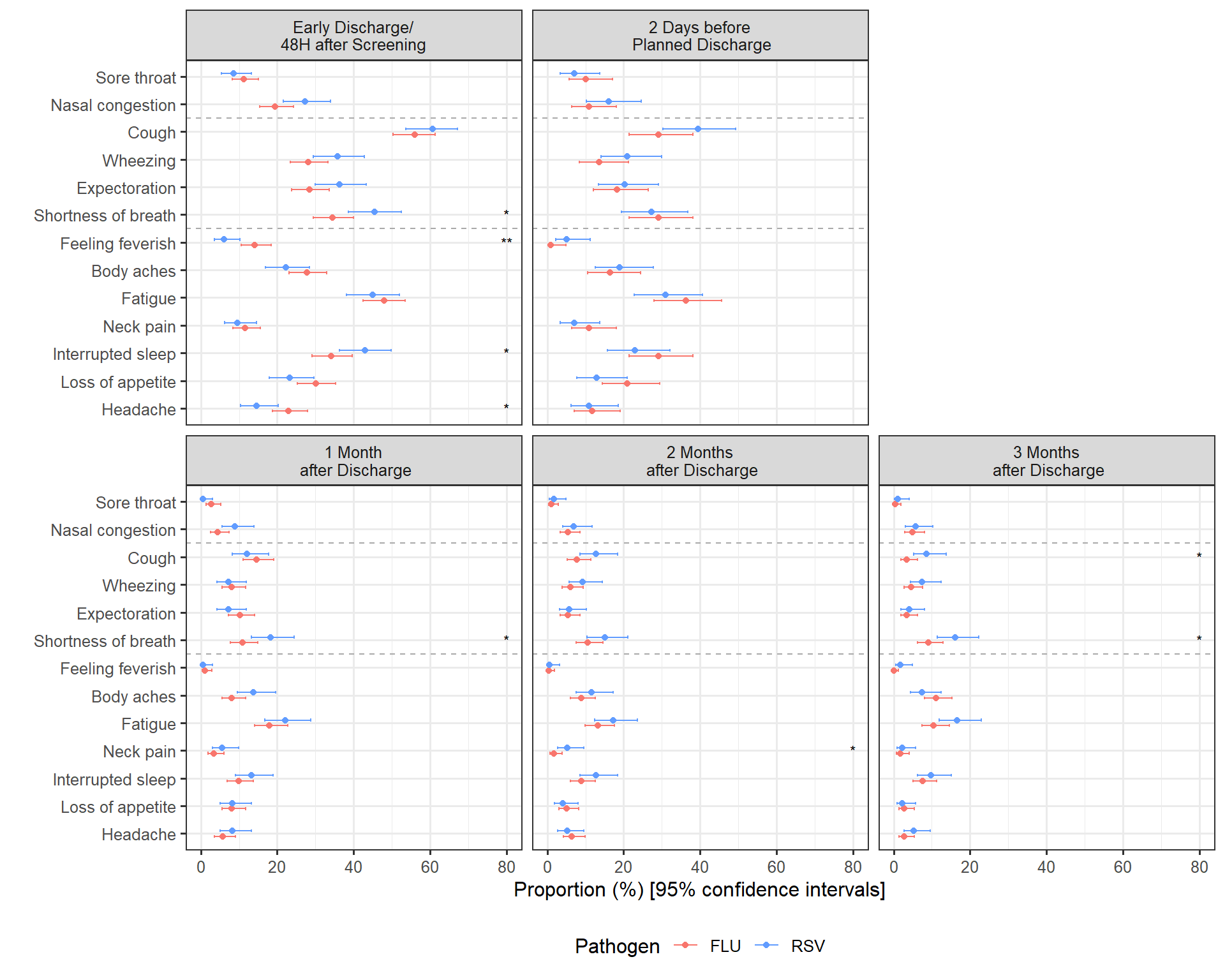
*

FLU: influenza; RSV: Respiratory Syncytial Virus.

95% Confidence intervals calculated using Wilson’s method. p-values based on Fisher-exact test: * p < 0.05; ** p < 0.01; *** p < 0.001

**Table 2 Mean RiiQ^TM^ Domain Score by Pathogen**

|  | **Influenza**  **(n=366)** | **RSV**  **(n=238)** | **hMPV**  **(n=100)** | **Total**  **(n=704)** |
| --- | --- | --- | --- | --- |
| **Lower respiratory tract symptoms** | | | | |
| Early discharge/48H after enrollment (n) | 313 | 198 | 88 | 599 |
| Mean (SE) | 1.18 (0.04) | 1.36 (0.05) | 1.42 (0.07) | 1.27 (0.03) |
| P-value^a^ |  |  |  | 0.001 |
| 2 Days before discharge (n) | 110 | 99 | 39 | 251 |
| Mean (SE) | 0.88 (0.06) | 1.02 (0.07) | 0.90 (0.10) | 0.94 (0.04) |
| P-value^a^ |  |  |  | 0.302 |
| 1 Month post-discharge | 295 | 181 | 81 | 562 |
| Mean (SE) | 0.47 (0.03) | 0.47 (0.04) | 0.40 (0.05) | 0.46 (0.02) |
| P-value^a^ |  |  |  | 0.694 |
| 2 Month post-discharge | 294 | 173 | 78 | 550 |
| Mean (SE) | 0.34 (0.03) | 0.46 (0.04) | 0.37 (0.06) | 0.38 (0.02) |
| P-value^a^ |  |  |  | 0.035 |
| 3 Month post-discharge | 287 | 174 | 69 | 535 |
| Mean (SE) | 0.26 (0.03) | 0.40 (0.04) | 0.36 (0.07) | 0.32 (0.02) |
| P-value^a^ |  |  |  | 0.007 |
| **Upper respiratory tract symptoms** | | | | |
| Early discharge/48H after enrollment | 313 | 198 | 88 | 599 |
| Mean (SE) | 0.62 (0.04) | 0.70 (0.05) | 0.69 (0.07) | 0.65 (0.03) |
| P-value^a^ |  |  |  | 0.068 |
| 2 Days before discharge | 110 | 99 | 39 | 251 |
| Mean (SE) | 0.42 (0.05) | 0.45 (0.06) | 0.35 (0.09) | 0.42 (0.04) |
| P-value^a^ |  |  |  | 0.558 |
| 1 Month post-discharge | 295 | 181 | 82 | 563 |
| Mean (SE) | 0.21 (0.03) | 0.24 (0.03) | 0.22 (0.05) | 0.22 (0.02) |
| P-value^a^ |  |  |  | 0.119 |
| 2 Month post-discharge | 294 | 173 | 78 | 550 |
| Mean (SE) | 0.16 (0.02) | 0.23 (0.03) | 0.18 (0.05) | 0.19 (0.02) |
| P-value^a^ |  |  |  | 0.166 |
| 3 Month post-discharge | 287 | 174 | 69 | 535 |
| Mean (SE) | 0.15 (0.02) | 0.18 (0.03) | 0.17 (0.04) | 0.16 (0.02) |
| P-value^a^ |  |  |  | 0.190 |
| **Respiratory symptoms** | | | | |
| Early discharge/48H after enrollment | 313 | 198 | 88 | 599 |
| Mean (SE) | 0.99 (0.04) | 1.14 (0.05) | 1.18 (0.06) | 1.07 (0.03) |
| P-value^a^ |  |  |  | 0.001 |
| 2 Days before discharge | 110 | 100 | 39 | 252 |
| Mean (SE) | 0.72 (0.05) | 0.83 (0.06) | 0.71 (0.09) | 0.77 (0.04) |
| P-value^a^ |  |  |  | 0.369 |
| 1 Month post-discharge | 295 | 181 | 82 | 563 |
| Mean (SE) | 0.38 (0.03) | 0.40 (0.03) | 0.34 (0.04) | 0.38 (0.02) |
| P-value^a^ |  |  |  | 0.470 |
| 2 Month post-discharge | 294 | 173 | 78 | 550 |
| Mean (SE) | 0.28 (0.02) | 0.38 (0.04) | 0.31 (0.05) | 0.32 (0.02) |
| P-value^a^ |  |  |  | 0.017 |
| 3 Month post-discharge | 287 | 174 | 69 | 535 |
| Mean (SE) | 0.22 (0.02) | 0.33 (0.03) | 0.29 (0.05) | 0.27 (0.02) |
| P-value^a^ |  |  |  | 0.005 |
| **Systemic symptoms** | | | | |
| Early discharge/48H after enrollment | 313 | 198 | 87 | 598 |
| Mean (SE) | 0.86 (0.04) | 0.79 (0.04) | 0.85 (0.06) | 0.83 (0.03) |
| P-value^a^ |  |  |  | 0.598 |
| 2 Days before discharge | 110 | 100 | 39 | 252 |
| Mean (SE) | 0.59 (0.05) | 0.55 (0.05) | 0.52 (0.08) | 0.56 (0.03) |
| P-value^a^ |  |  |  | 0.819 |
| 1 Month post-discharge | 295 | 181 | 82 | 563 |
| Mean (SE) | 0.30 (0.02) | 0.35 (0.03) | 0.35 (0.05) | 0.32 (0.02) |
| P-value^a^ |  |  |  | 0.065 |
| 2 Month post-discharge | 294 | 173 | 78 | 550 |
| Mean (SE) | 0.24 (0.02) | 0.31 (0.03) | 0.30 (0.05) | 0.27 (0.02) |
| P-value^a^ |  |  |  | 0.044 |
| 3 Month post-discharge | 287 | 174 | 69 | 535 |
| Mean (SE) | 0.22 (0.02) | 0.25 (0.03) | 0.28 (0.05) | 0.23 (0.02) |
| P-value^a^ |  |  |  | 0.308 |

hMPV: Human Metapneumovirus; RSV: Respiratory Syncytial Virus, SE: Standard error.

^a^ p-value based on Kruskal-Wallis testing for differences between Influenza, RSV and hMPV participants.

**Table 3 Mean RiiQ^TM^ Domain Score by Presence of Core Risk Factor (CRF)**

|  | **CRF (+)**  **(n=566)** | **CRF (-)**  **(n=138)** | **Total**  **(n=704)** |
| --- | --- | --- | --- |
| Lower Respiratory Tract Symptoms | | | |
| Early discharge/48H after enrollment (n) | 472 | 127 | 599 |
| Mean (SE) | 1.34 (0.03) | 1.04 (0.06) | 1.27 (0.03) |
| P-value ^a^ |  |  | <0.001 |
| 2 Days before discharge (n) | 205 | 43 | 248 |
| Mean (SE) | 0.99 (0.05) | 0.69 (0.08) | 0.94 (0.04) |
| P-value ^a^ |  |  | 0.006 |
| 1 Month post-discharge (n) | 441 | 116 | 557 |
| Mean (SE) | 0.50 (0.03) | 0.32 (0.04) | 0.46 (0.02) |
| P-value ^a^ |  |  | 0.007 |
| 2 Month post-discharge (n) | 431 | 114 | 545 |
| Mean (SE) | 0.41 (0.03) | 0.27 (0.04) | 0.38 (0.02) |
| P-value ^a^ |  |  | 0.007 |
| 3 Month post-discharge (n) | 427 | 103 | 530 |
| Mean (SE) | 0.36 (0.03) | 0.15 (0.03) | 0.32 (0.02) |
| P-value ^a^ |  |  | <0.001 |
| **Upper respiratory tract symptoms** | | | |
| Early discharge/48H after enrollment (n) | 472 | 127 | 599 |
| Mean (SE) | 0.65 (0.03) | 0.69 (0.07) | 0.65 (0.03) |
| P-value ^a^ |  |  | 0.938 |
| 2 Days before discharge (n) | 205 | 43 | 248 |
| Mean (SE) | 0.43 (0.04) | 0.37 (0.09) | 0.42 (0.04) |
| P-value ^a^ |  |  | 0.246 |
| 1 Month post-discharge (n) | 442 | 116 | 558 |
| Mean (SE) | 0.24 (0.02) | 0.17 (0.03) | 0.22 (0.02) |
| P-value ^a^ |  |  | 0.378 |
| 2 Month post-discharge (n) | 431 | 114 | 545 |
| Mean (SE) | 0.19 (0.02) | 0.17 (0.03) | 0.19 (0.02) |
| P-value ^a^ |  |  | 0.528 |
| 3 Month post-discharge (n) | 427 | 103 | 530 |
| Mean (SE) | 0.18 (0.02) | 0.11 (0.03) | 0.16 (0.02) |
| P-value ^a^ |  |  | 0.080 |
| **Respiratory symptoms** | | | |
| Early discharge/48H after enrollment (n) | 472 | 127 | 599 |
| Mean (SE) | 1.11 (0.03) | 0.92 (0.06) | 1.07 (0.03) |
| P-value ^a^ |  |  | 0.002 |
| 2 Days before discharge (n) | 206 | 43 | 249 |
| Mean (SE) | 0.80 (0.04) | 0.58 (0.07) | 0.77 (0.04) |
| P-value ^a^ |  |  | 0.014 |
| 1 Month post-discharge (n) | 442 | 116 | 558 |
| Mean (SE) | 0.41 (0.02) | 0.27 (0.03) | 0.38 (0.02) |
| P-value ^a^ |  |  | 0.007 |
| 2 Month post-discharge (n) | 431 | 114 | 545 |
| Mean (SE) | 0.34 (0.02) | 0.24 (0.03) | 0.32 (0.02) |
| P-value ^a^ |  |  | 0.015 |
| 3 Month post-discharge (n) | 427 | 103 | 530 |
| Mean (SE) | 0.30 (0.02) | 0.13 (0.03) | 0.27 (0.02) |
| P-value ^a^ |  |  | <0.001 |
| **Systemic symptoms** | | | |
| Early discharge/48H after enrollment (n) | 471 | 127 | 598 |
| Mean (SE) | 0.85 (0.03) | 0.75 (0.06) | 0.83 (0.03) |
| P-value ^a^ |  |  | 0.034 |
| 2 Days before discharge (n) | 206 | 43 | 249 |
| Mean (SE) | 0.58 (0.04) | 0.47 (0.08) | 0.56 (0.03) |
| P-value ^a^ |  |  | 0.196 |
| 1 Month post-discharge (n) | 442 | 116 | 558 |
| Mean (SE) | 0.34 (0.02) | 0.27 (0.03) | 0.32 (0.02) |
| P-value ^a^ |  |  | 0.028 |
| 2 Month post-discharge (n) | 431 | 114 | 545 |
| Mean (SE) | 0.28 (0.02) | 0.22 (0.03) | 0.27 (0.02) |
| P-value ^a^ |  |  | 0.046 |
| 3 Month post-discharge (n) | 427 | 103 | 530 |
| Mean (SE) | 0.25 (0.02) | 0.17 (0.03) | 0.23 (0.02) |
| P-value ^a^ |  |  | 0.015 |

CRF: Core Risk Factor; SE: Standard error.
^a^ p-value based on Kruskal-Wallis testing for differences between CRF(+) and CRF(-) participants.

**Figure 2** **EQ-5D-5L VAS Score over Time by Pathogen**


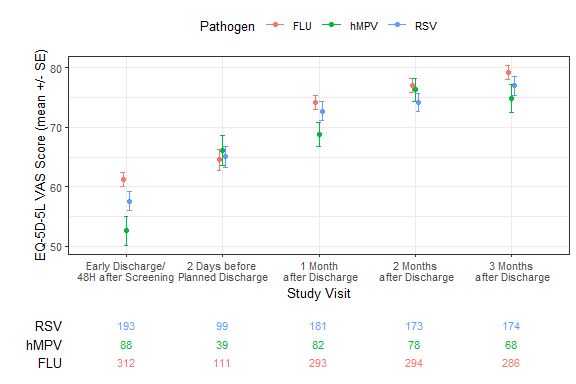


Co-infections (N=5) are excluded

**Figure 3 EQ-5D-5L VAS Score over Time by Presence of Core Risk Factors**


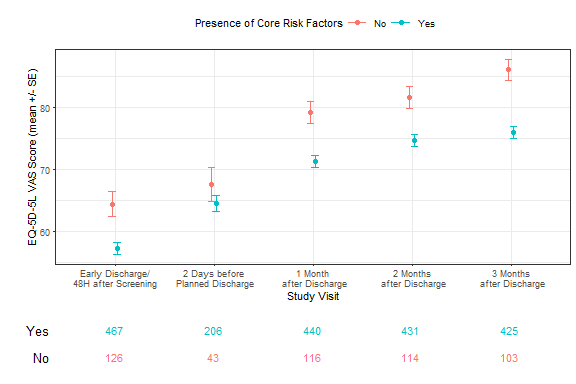


Co-infections (N=5) are excluded

**Figure 4 Association Between RiiQ^TM^ Score and EQ-5D-5L VAS Score Over Time**
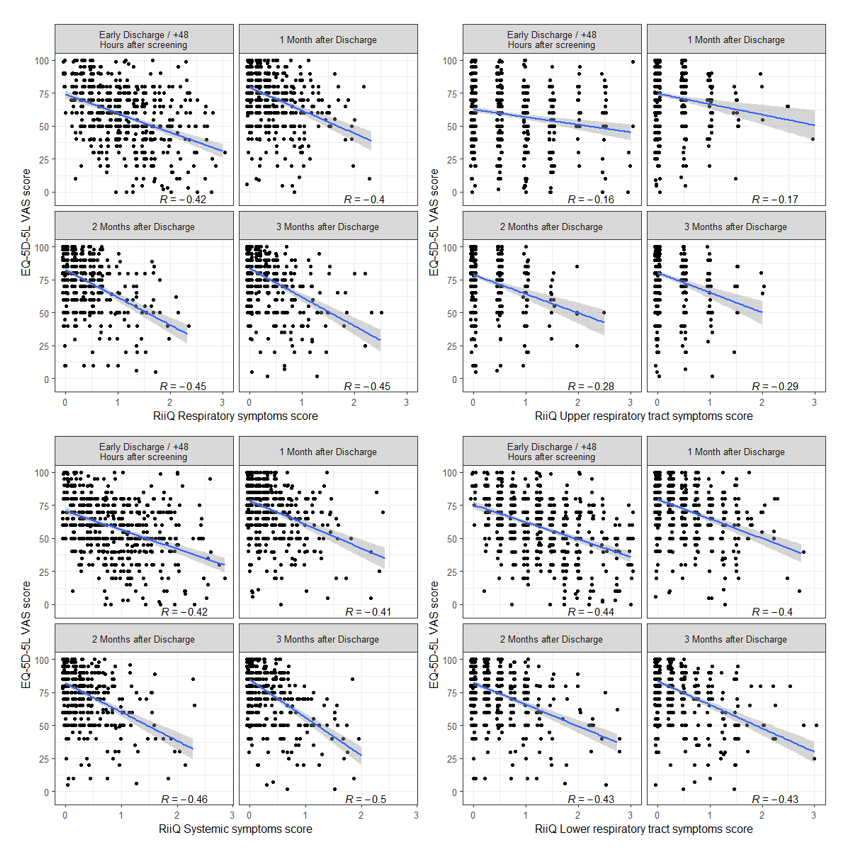


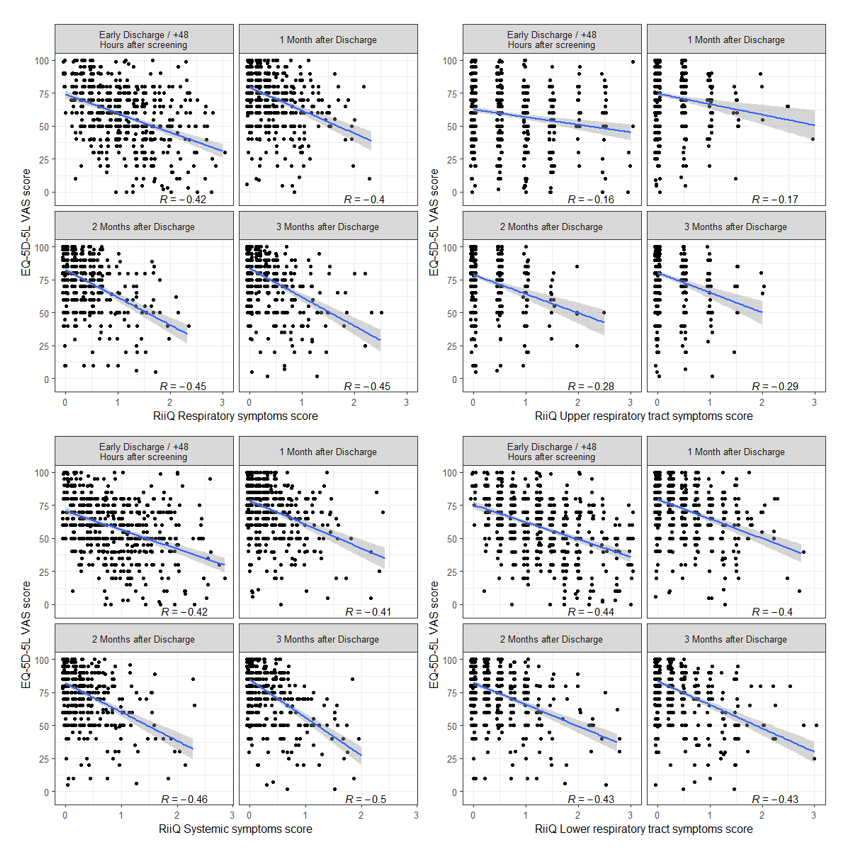


EQ-5D-5L VAS=EuroQol 5 Dimensions 5 Levels Visual Analogue Scale; RiiQ^TM^=Respiratory Intensity and Impact Questionnaire.

Small random jitter is applied to the data points to allow for a clearer view on the data points. Spearman correlation coefficient (R) is presented. A linear regression line (with 95% confidence interval) is added to aid visualization.

Co-infections (N=5) are excluded
